# Cold-induced vasodilation during sequential immersions of the hand

**DOI:** 10.1007/s00421-023-05304-2

**Published:** 2023-10-21

**Authors:** Rebecca S. Weller, Hein A. Daanen, Rebecca J. McClintock, Nicholas A. Roberts, Timothy L. Dunn, Douglas M. Jones

**Affiliations:** 1 Naval Health Research Center, 140 Sylvester Rd, San Diego, CA USA; 2 Leidos, Inc., San Diego, CA USA; 3 Vrije Universiteit Amsterdam, Amsterdam, The Netherlands; 4 Marine Corps Mountain Warfare Training Center, Bridgeport, CA USA

**Keywords:** Hunting reaction, Frostbite prevention, Rewarming, Finger skin temperature

## Abstract

A common practice for those operating in cold environments includes repetitive glove doffing and donning to perform specific tasks, which creates a repetitive cycle of hand cooling and rewarming. This study aimed to determine the influence of intraday repeated hand cooling on cold-induced vasodilation (CIVD), sympathetic activation, and finger/hand temperature recovery. Eight males and two females (mean ± SD age: 28 ± 5 year; height: 181 ± 9 cm; weight: 79.9 ± 10.4 kg) performed two 30-min hand immersions in cold (4.3 ± 0.92 °C) water in an indoor environment (18 °C). Both immersions (Imm1; Imm2) were performed on the same day and both allowed for a 10-min recovery. CIVD components were calculated for each finger (index, middle, ring) during each immersion. CIVD onset time (index, *p* = 0.546; middle, *p* = 0.727; ring, *p* = 0.873), minimum finger temperature (index, *p* = 0.634; middle, *p* = 0.493; ring, *p* = 0.575), and mean finger temperature (index, *p* = 0.986; middle, *p* = 0.953; ring, *p* = 0.637) were all similar between immersions. Recovery rates generally demonstrated similar responses as well. Findings suggest that two sequential CIVD tests analyzing the effect of prior cold exposure of the hand does not impair the CIVD response or recovery. Such findings appear promising for those venturing into cold environments where hands are likely to be repeatedly exposed to cold temperatures.

## Introduction

For those working, operating, and recreating in cold environments, exposure to low ambient temperatures can limit optimal performance and induce localized cold injuries with a presence or absence of tissue freezing (Haman et al. 2022; Havenith et al. 1995; Norrbrand et al. 2020). During the initial moments of whole-body cold stress, sympathetic activation induces vasoconstriction, shunting blood from peripheral extremities to the core region of the body to minimize heat loss and maintain warmth of vital organs (Tyler et al. 2015). This trade-off in blood flow results in colder hands and feet. When hand temperatures fall below 15 °C, a sharp decrement in dexterity is observed, and below 8 °C, nerve conduction is impaired, resulting in tactile sensitivity loss (Havenith et al. 1995; Heus et al. 1995). To recover function, rewarming strategies, such as donning additional thermal protective layers, endogenous heat production (e.g., exercise), and exogenous rewarming (e.g., commercial heat packs, heaters, body-to-body contact, and other sources of heat), may be necessary (Jones et al. 2020). Without timely rewarming intervention, hands and feet may continue to lose heat until tissue freezes, resulting in frostbite (Imray et al. 2009).

Cold-water immersion is generally used to investigate the effects of cold on the hands and feet thus avoiding the risk of cold injury. When hands or feet are exposed to cold, a local thermoregulatory reaction termed “cold-induced vasodilation (CIVD)” or “the hunting reaction” occurs, wherein arterio-venous anastomoses (AVAs) dilate in an oscillatory pattern to provide hands and feet with transient episodes of warm blood (Daanen 2003). CIVD has been investigated since 1930, when Sir Thomas Lewis first described the effect after immersing the fingertip in 0 °C water (Lewis 1930). Strong CIVD responses may help maintain dexterity and tissue temperature of the fingers and toes during cold exposure, and may also delay the onset, or prevent the occurrence, of frostbite (Wilson and Goldman 1970; Daanen and Van der Struijs 2005). CIVD is often assessed by measuring finger skin temperature or skin blood flow during a 30-min hand immersion in cold water and is characterized by several components: (1) vasodilation onset time (Δt_onset_), (2) mean finger skin temperature (T_mean_), (3) minimum finger skin temperature (T_min_), (4) magnitude of response (increase in finger temperature), and (5) frequency of response (how often vasodilation occurs); (Daanen 2003; Daanen and van der Struijs 2005; O’Brien 2005; Tsoutsoubi et al. 2022). These CIVD elements play important roles in how well hand and foot temperatures are maintained and recover in cold environments.

A common practice for those operating in cold environments includes repetitive glove doffing and donning to perform specific tasks requiring a high degree of dexterity, such as equipment operation, weapon handling, medical care, and survival skills (Sullivan-Kwantes et al. 2021). Frequent glove doffing and donning creates a repetitive cycle of hand cooling and rewarming, yet limited investigations have been performed on this topic as it relates to CIVD and hand temperature recovery. CIVD responses have been evaluated with repeated hand immersions (one each day) performed over consecutive days (Daanen et al. 2012; Geurts et al. 2006a; Mekjavic et al. 2008; O’Brien 2005), but the intraday effects on repetitive hand cooling and recovery have not been investigated. Critically, intraday repetitive hand cooling is most representative of the cyclical behavior observed with glove doffing and donning in the cold.

This study aimed to determine the influence of two sequential intraday hand immersions in cold water on CIVD and finger/hand temperature recovery. We hypothesized that a second immersion, occurring within minutes after the first, would impair the CIVD response, possibly due to attenuated vasodilation, and limit optimal recovery. Evidence gained from this effort provides critical insights into the relationship between intraday repetitive hand cooling and risk for cold injury.

## Methods

### Participants

Eight male and two female active-duty personnel (mean ± SD age: 28 ± 5 yr; height: 181 ± 9 cm; weight: 79.9 ± 10.4 kg) participating in a military medical training exercise volunteered for the study. In compliance with the Institutional Review Board of the Naval Health Research Center (Protocol # NHRC.2021.0002), all participants provided voluntary informed consent and Health Insurance Portability and Accountability Act authorization. All participants were considered “fit for full duty,” based on the requirements to participate in the medical training. Additionally, participants were asked to refrain from caffeine or nicotine consumption during their study participation.

### Setup and participant instrumentation

**Fig. 1:**
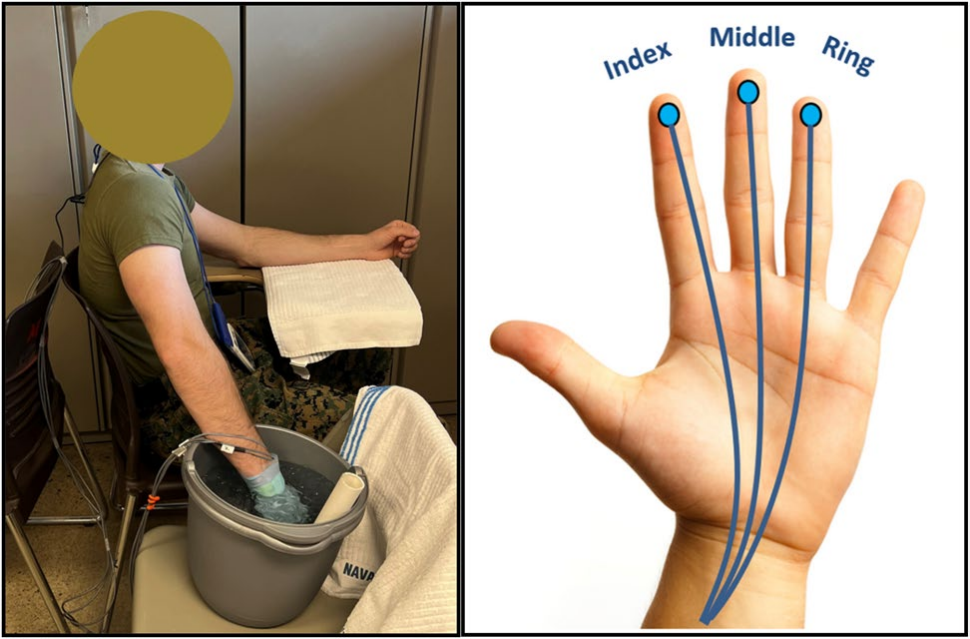
(Left) photo of participant performing cold-water hand immersion. (Right) placement of thermistors for finger skin temperature collection during immersion and rewarming Photo Credit: Naval Health Research Center

Approximately 6 h prior to testing, participants ingested a temperature capsule (BodyCap, Saint-Clair, France) for core temperature (T_c_) measurement. T_c_ was measured to confirm an absence of significant fluctuations in deep body temperature, which could potentially influence CIVD responses. Participants wore their standard-issued battle dress uniforms, consisting of socks, boots, trousers, and blouse during all test procedures. They were instrumented with a Polar heart rate (HR) monitor and chest strap (Polar Electro©, Bethpage, NY) to continuously monitor cardiovascular and sympathetic responses to cold-water hand immersion. Skin temperature thermistors were attached to the skin of the index, middle, and ring fingers in the middle of the palmar side of the distal phalanx of the right hand (Deban Enterprises Inc., Dayton, OH) to evaluate CIVD responses and recovery performance (i.e., spontaneous finger rewarming); (Fig. 1). The finger skin temperatures were measured with Surface Temperature sensor (Model 409A) thermocouples (8.75 mm in diameter) probes (Deban Enterprises Inc., Dayton, OH). Additionally, a Thermocron iButton temperature sensor (iButtonLink Technology, Whitewater, WI) was placed on the posterior aspect of the right hand to measure hand skin temperature (T_hand_) during immersion and recovery. Participants were then provided time to become familiar with hand pain sensation (P_hand_), hand thermal sensation (TS_hand_), and whole-body thermal sensation (TS_body_) scales prior to their use. The pain scale for the hand ranged from 0 (No pain at all) to 10 (maximal pain conceivable). Ratings for TS_hand_ and TS_body_ ranged from + 4 (very hot) to 0 (neutral) to – 4 (very cold). A nitrile medical exam glove (thickness of 0.08 mm) was then donned and lightly taped at the wrist, careful not to constrict blood flow, to ensure that sensors did not come into direct contact with water during hand immersion. During instrumentation, participants sat for a minimum of 20–30 min before the first cold-water hand immersion.

### Hand immersion tests

**Fig. 2:**
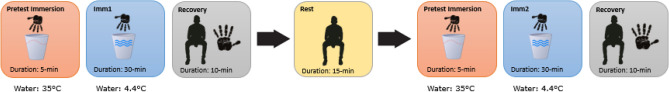
Schematic detailing repeated cold-water hand immersion and recovery protocol. All testing was performed in ambient room temperature (18 °C)

Participants performed two 30-min hand immersions in cold (4.3 ± 0.92 °C) water in an indoor environment (18 °C). Both immersions were performed on the same day, and both allowed for 10 min of recovery following each immersion. There was a 15-min period of quiet sitting between immersions (from the end of recovery of the first immersion to the start of the second immersion) (Fig. 2). Participants started the hand immersion procedure by placing their right hand in warm (35 °C) water for 5 min to standardize hand temperature prior to the start of cold-water immersion (Tsoutsoubi et al. 2022; Tyler et al. 2015). Immediately after hand immersion in warm water, participants placed their hand into cold water (to the level of the ulnar styloid process) to begin the first cold immersion. The water temperature was closely monitored and stirred every 2 min, and ice was added to ensure that water temperature remained at 4.3 ± 0.92 °C °C. T_hand_ and skin temperatures for index (T_index_), middle (T_middle_), and ring (T_ring_) fingers were recorded each minute, while subjective measurements of P_hand_, TS_hand_, and TS_body_ were recorded every two minutes. Participants kept their hand immersed in cold water for the entire 30-min immersion. Cognitive testing (auditory simple reaction time) was performed during immersion, but these data are not presented in this manuscript. For general awareness, participants wore headphones to listen for auditory beeps, which were presented at randomized interstimulus intervals. They had to respond with the word “go” as fast as possible each time they heard a beep. The stimulus onset time was subtracted from and response time to evaluate auditory reaction time.

### Recovery procedures

At the conclusion of the first 30-min hand immersion in cold water (Imm1), participants removed their hand from water and the researcher removed the nitrile glove. While remaining seated, participants rested their hand flat on a table for the entirety of the 10-min recovery to allow for spontaneous rewarming. P_hand_, TS_hand_, and TS_body_ were recorded every 2 min during recovery, while T_hand_ and finger skin temperatures (T_index_, T_middle_, T_ring_) were recorded each minute. After the 10-min recovery, participants sat quietly for 15 min before starting the second immersion (Imm2). Imm2 followed the same procedures as Imm1, beginning with hand immersion in warm water for 5 min.

### CIVD components

**Fig. 3:**
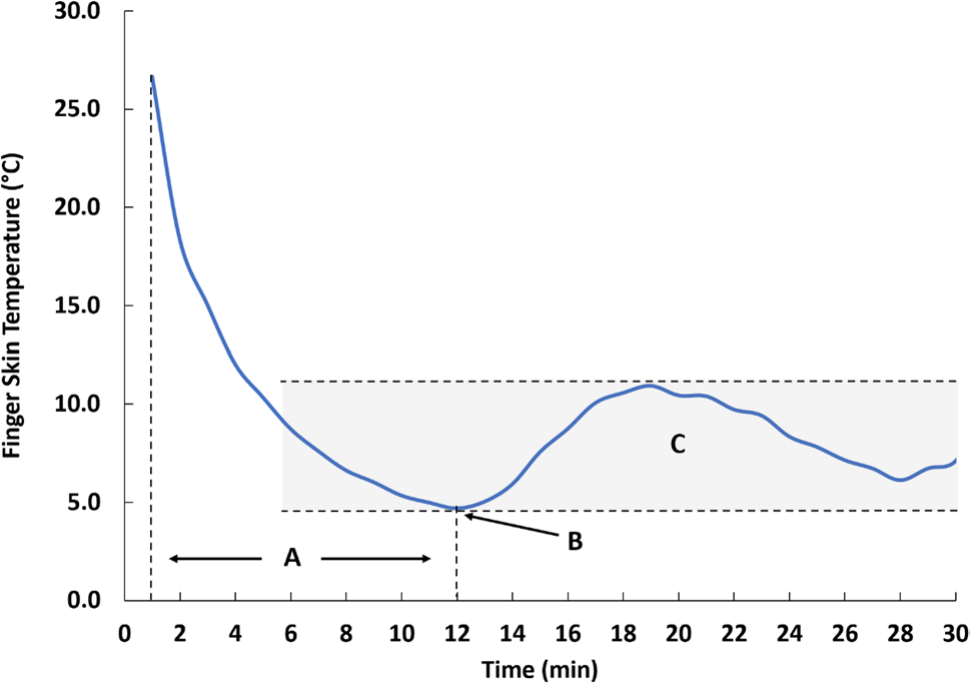
Example of finger temperature response to cold-water immersion of the hand which includes CIVD components, where CIVD onset time **A** is the amount of time from the start of immersion to the onset of vasodilation; T_min_ **B** is the minimum finger temperature, and T_mean_ **C** is the mean finger temperature calculated from minutes 5 to 30 of immersion

CIVD components (Fig. 3) were calculated for each immersion and each finger (index, middle, ring) using the methods described by Daanen (Daanen 2003) and included: minimum finger skin temperature (T_min_; the lowest finger skin temperature just before the onset of CIVD), CIVD onset time (Δt_onset_; the time from start of immersion to T_min_), and mean finger skin temperature (T_mean_; the average skin temperature during the cold-water immersion excluding the initial 5 min of cold-water immersion). The criterion for CIVD occurrence was defined as an uninterrupted increase in finger temperature > 0.5 °C (Cheung 2015).

### Data analysis

Repeated-measures analyses of variance (ANOVA) were used to compare Δt_onset_, T_min_, and T_mean_ between Imm1 and Imm2 for each of the three fingers (index, middle, ring). T_hand_ (immersion average), T_c_ (immersion average), HR (average of the first 10 min of immersion), P_hand_ (immersion average), TS_hand_ (immersion average), and TS_body_ (immersion average) were also analyzed using repeated-measures ANOVA. For recovery, T_index_, T_middle_, T_ring_, and T_hand_ recovery rates (i.e., spontaneous rewarming), as well as P_hand_ (recovery average), TS_hand_ (recovery average), and TS_body_ (recovery average) were compared between the first (Rec1) and second (Rec2) recovery periods using repeated-measures ANOVA. The average of all three fingers skin temperatures and hand temperatures during the 5 min warm-water immersion (35 °C) was analyzed using repeated-measures ANOVA to confirm standardized starting skin temperatures prior to each immersion. Data were analyzed to confirm normal distribution using a test of homogeneity of variance. Multiple comparisons were corrected for using the Bonferroni correction. All analyses were performed using Statistical Package for the Social Sciences (SPSS Inc. ®, version 25, Chicago, IL). Data are presented as mean ± SD with significance level set at * p* < 0.05.

## Results

### Hand immersion in cold water

In each immersion, all participants demonstrated a CIVD response, evidenced by an uninterrupted 0.5 °C increase in finger temperature after the drop to T_min_. There were no differences in finger temperature between the two 5-min warm-water immersions for the average of all 3 fingers (Imm1: 27.82 + 2.6 °C, Imm2: 28.62 + 2.9 °C; *p* = 0.066) or the average hand skin temperature (Imm1: 30.35 + 1.4 °C, Imm2: 27.94 + 1.3 °C; *p* = 0.006) during the 5 min immersion in warm 35 °C water, thus confirming similar starting temperatures. Although all participants met the minimum threshold for CIVD, no differences were observed between Imm1 and Imm2 for any of the CIVD components (Δt_onset_, T_min_, T_mean_) or number of CIVD waves (Imm1: 1.0 + 0.4, Imm2: 1.0 + 0.6; *p* = 0.423). This finding was true for each finger (index, middle, ring); (Table 1). Contrary to the similar temperature responses observed in the fingers between immersions, T_hand_ was colder during Imm2 compared with Imm1 (Imm1: 12.4 ± 2.2 °C, Imm2: 10.4 ± 2.1 °C,* p* =  < 0.01). We were able to confirm that no significant changes in deep body temperature occurred, as T_c_ was not different between immersions (Imm1: 37.1 ± 0.2 °C, Imm2: 37.0 ± 0.3 °C; *p* = 0.054). A possible acute sympathetic habituation response occurred, as HR (mean of first 10 min of immersion) was lower during Imm2 compared with Imm1 (Imm1: 76 ± 12 bpm, Imm2: 68 ± 9 bpm; *p* < 0.01). Although T_hand_ demonstrated lower temperature on Imm2, P_hand_ (Imm1: 2.7 ± 1.6, Imm2: 2.5 ± 1.2, *p* = 0.487) and TS_hand_ (Imm1: − 2.8 ± 0.8, Imm2: − 1.9 ± 1.9, *p* = 0.199) were not different between immersions. TS_Body_ (Imm1: − 0.8 ± 0.9, Imm2: − 0.4 ± 1.2, *p* = 0.165) was also not different between immersions (Table 2).

**Table 1:** Mean ± SD CIVD responses for index, middle, and ring fingers between Imm1 and Imm2

	Imm1	Imm2	Sig.
Δt_onset_ (min)			
Index	12.1 ± 2.9	13.2 ± 4.3	*p* = 0.546
Middle	12.2 ± 3.6	11.6 ± 3.4	*p* = 0.727
Ring	13.1 ± 7.1	12.6 ± 8.8	*p* = 0.873
T_min_ (°C)			
Index	6.3 ± 1.7	6.5 ± 1.4	*p* = 0.634
Middle	5.9 ± 1.8	6.3 ± 1.4	*p* = 0.493
Ring	5.9 ± 1.6	6.2 ± 1.3	*p* = 0.575
T_mean_ (°C)			
Index	8.8 ± 1.4	8.8 ± 0.6	*p* = 0.986
Middle	8.6 ± 1.2	8.6 ± 0.6	*p* = 0.953
Ring	7.8 ± 1.4	7.6 ± 1.4	*p* = 0.637

No differences were observed between immersions for any measurement

**Table 2:** Mean ± SD perceptual responses for each immersion (Imm1, Imm2) and each recovery (Rec1, Rec2)

	Imm1	Imm2	Rec1	Rec2
P_hand_	2.7 ± 1.6	2.5 ± 1.2	1.3 ± 1.4	0.1 ± 0.2*
TS_Hand_	− 2.8 ± 0.8	− 1.9 ± 1.9	− 1.1 ± 1.1	− 0.6 ± 1.3
TS_Body_	− 0.8 ± 0.9	− 0.4 ± 1.2	− 0.03 ± 0.9	− 0.2 ± 1.2

Asterisk (*) indicates significant difference between recovery periods (*p* < 0.05)

### Recovery

Spontaneous rewarming occurred in the fingers following each immersion during the 10-min recovery periods. Recovery rates for T_index_ (Rec1: 1.3 ± 0.2 °C/min, Rec2: 1.1 ± 0.2 °C/min, *p* = 0.051) and T_middle_ (Rec1: 1.2 ± 0.3 °C/min, Rec2: 1.0 ± 0.3 °C/min, *p* = 0.166) were similar between Rec1 and Rec2. T_ring_, however, recovered faster in Rec1 compared with Rec2 (Rec1: 1.2 ± 0.3 °C/min, Rec2: 1.0 ± 0.3 °C/min, *p* = 0.018). Despite a lower T_hand_ observed during Imm2, T_hand_ recovery was similar between each recovery period (Rec1: 0.7 ± 0.2 °C/min, Rec2: 0.6 ± 0.1 °C/min, *p* = 0.058). Participants perceived less pain during Rec2 compared with Rec1 (Rec1: 1.3 ± 1.4, Rec2: 0.1 ± 0.2; *p* = 0.019), whereas thermal sensation ratings for TS_hand_ (Rec1: − 1.1 ± 1.1, Rec2: − 0.6 ± 1.3, *p* = 0.118) and TS_Body_ (Rec1: − 0.03 ± 0.9, Rec2: − 0.2 ± 1.2, *p* = 0.415) were similar between recovery periods (Table 2).

## Discussion

This study aimed to investigate the influence of two sequential intraday hand immersions in cold water on CIVD responses, sympathetic activity, and spontaneous finger rewarming. The primary outcome suggests that contrary to our hypothesis, consecutive hand immersion in cold water does not impair any elements of CIVD, including Δt_onset_, T_min_, and T_mean_. However, we did observe lower T_hand_ during Imm2. A secondary finding suggests that T_hand_ and finger temperature during recovery is not significantly affected by a second immersion. Although these findings demonstrate generally unimpaired thermoregulation in the distal extremities, there may be a presence of attenuated sensory activation which may alter behavioral thermoregulation, as evidenced by lower T_hand_ coupled with invariable pain sensation.

It is well understood that cold stress excites cold-temperature receptors imbedded in the skin, causing subsequent activation of the sympathetic nervous system, release of norepinephrine, and activation of α-adrenergic receptors (Alba et al 2019). This cascade of events ultimately leads to an effector response on the blood vessels, causing them to vasoconstrict. The paradoxical vasodilation that occurs during cold stress has not been definitively described, yet several mechanistic hypotheses have been summarized in a review by Daanen (Daanen 2003). The two most likely hypotheses put forward for the mechanism of CIVD include: (1) vasodilatory agonists (released during cooling) interact with AVAs and other cutaneous vessels to cause transient vasodilation (Aschoff 1944), and (2) attenuation of norepinephrine (or cold-mediated nervous block) in the minutes following cold stress allows for episodic vasodilation to occur (Gardner and Webb 1986; Freedman et al. 1992). The second hypothesis could explain why vasoconstriction initially occurs (sympathetic activation), but nerve block (caused by cold nerves as tissue temperature falls) prevents neural signals from being transmitted to smooth muscle, thus allowing for relaxation and vasodilation. However, additional investigations are required to elucidate the exact mechanisms of CIVD.

Similar CIVD responses between Imm1 and Imm2 suggest that, whichever the true underlying causes of CIVD are, they remain unimpaired during subsequent intraday cold exposure. We did observe a reduced HR during the second immersion, possibly related to elevated anticipatory anxiety occurring during the first immersion which was not present during the second immersion. However, it would be expected that a lower HR, and thus a reduced sympathetic response, would have produced dissimilar CIVD results between Imm1 and Imm2, since norepinephrine plays an important role in CIVD. It is possible that changes in the sympathetic response, although detected on a systemic level, did not reflect the localized effects observed at the distal extremities. It has also been established that repeated cold exposures attenuate sympathetic activation (Leppaluoto et al. 2001). Another factor reported to alter CIVD is change in deep body temperature. We did not observe any changes in core temperature between immersions, which supports the similar CIVD responses between Imm1 and Imm2. However, it should be noted that minor decreases in core temperature weaken CIVD responses, and when core temperature becomes too low, CIVD is completely abolished (Flouris et al. 2008; Flouris and Cheung 2009). The reverse is also supported, wherein small increases in core temperature enhance CIVD (Nielsen 1987; Takano 1989).

Sensations of pain and thermal sensation were obtained during immersion and recovery. Although CIVD remained unaltered between immersions, we did observe lower T_hand_ on the second immersion. This finding may be related to the specific locations of AVAs, which are mostly found in the fingertips and not in the more proximal regions of the hands (Bergersen et al. 1997; Walloe 2016). Thus, upon cold exposure, CIVD was occurring in the fingers, but not the hand. As a result, the hand became colder on the second immersion likely due to less heat retention in deeper tissues and structures of the hand where no AVAs are present. When this finding is combined with an unchanged pain rating of the hand, it might suggest acute pain desensitization on the second immersion. A loss of ability to detect associated changes in pain sensation with changes in T_hand_ could increase risk for peripheral cold injury, as people often use pain sensation as a threshold for behavioral response, such as the need to don gloves in a cold-weather environment. The issue of attenuated pain sensations associated with cold acclimation have been described previously, and it remains under debate whether a reduction in pain sensation is beneficial or simply elevates risk for cold-weather injuries (Geurts et al. 2006b).

Spontaneous rewarming following immersion was evaluated, as it is a critical factor in determining one’s ability to recover from cold stress. T_index_, T_middle_, and T_hand_ demonstrated similar recovery rates, but T_ring_ recovered slower after the second immersion. Although a 0.2 °C/min difference in recovery may appear underwhelming, this equates to a reduction in finger temperature of 2 °C at the end of recovery compared with other fingers with faster rewarming rates. This finding requires additional investigation, as it is unclear why only the ring finger was unable to rewarm at the rates observed in the other two fingers. P_hand_ was also attenuated during Rec2 compared with Rec1, despite similar recovery rates in T_hand_ and most fingers. This finding during recovery is problematic, as it is with immersion, as disassociations between pain sensation and finger and hand temperatures creates a situation where individuals may feel more recovered than they actually are, putting them at greater risk for peripheral cold injury.

Several study limitations must be acknowledged and integrated into the interpretation of study findings. First, this study was performed at 2100 m elevation, which is considered moderate altitude. Previous work suggests that CIVD may be impacted by higher altitudes (Daanen and van Ruiten 2000). Although we do not believe that this elevation had any significant impact on the repeated-measures design of our CIVD testing, we must acknowledge that the study was not conducted at sea level. Another limitation includes the simultaneous administration of cognitive testing (auditory simple reaction time) during each immersion and recovery. The inclusion of cognitive testing introduces a potential distracting effect, which could confound ratings of pain and thermal sensation (Enander 1987; Lin et al. 2022).Finally, individual factors that influence CIVD, such as diet and sleep, were not controlled for, and could have impacted normal CIVD responses (Daanen 2003). However, no food items were ingested during testing, which covered both immersions and recovery periods. Additionally, we must acknowledge that immersing the hand back in 35 °C water prior to the second cold-water hand immersion is not the same as putting a glove back on in the field. It is assumed that glove doffing and donning would oscillate between rewarming and cooling the hand and fingers. However, to maintain measurement consistency and control, standardizing starting temperatures prior to each immersion was critical. The size of the participants’ hand and fingers might also have affected skin temperature responses to the cold-water and rewarming rates (Jay and Havenith 2004). Future studies may consider analyzing and reporting finger anthropometrics.

Based on the finger skin temperature recordings obtained during this study, it appears that two sequential intraday hand immersions in cold water do not impair the CIVD response. Evidence also suggest that finger and hand temperature recovery rates are similar after consecutive hand immersions. Such findings appear promising for those venturing into cold environments where hands are likely to be repeatedly exposed to cold temperatures. However, additional investigation is warranted regarding the interplay between pain sensation and finger temperature during repeated cold exposure, as asynchronous changes in these factors may introduce risk for peripheral cold injury.

## Abbreviations

Δt_onset_: Vasodilation onset time
AVA: Arterio-venous anastomoses
CIVD: Cold-induced vasodilation
Imm1: First immersion
Imm2: Second immersion
P_hand_: Hand pain sensation
Rec1: First recovery
Rec2: Second recovery
SD: Standard deviation
T_c_: Core temperature
T_index_: Index finger temperature
T_middle_: Middle finger temperature
T_min_: Minimum finger temperature
T_mean_: Mean finger temperature
T_ring_: Ring finger temperature
TS_body_: Whole-body thermal sensation
TS_hand_: Hand thermal sensation
